# The need for sensory nutrition research in individuals with smell loss

**DOI:** 10.1016/j.nutos.2022.11.002

**Published:** 2022-11-11

**Authors:** Stephanie R. Hunter, Pamela H. Dalton

**Affiliations:** Monell Chemical Senses Center, Philadelphia, PA, USA

**Keywords:** Olfaction, Nutrition, Chronic disease, Diet quality, Flavor, Salt, Capsaicin

## Abstract

Millions of people will now suffer from long-term smell loss as a result of infection with the SARS-CoV-2 virus. Smell is an integral component of the flavor of foods, which is one of the primary drivers of ingestive behavior. When individuals lose their sense of smell, they find food to be less flavorful and less enjoyable, which negatively impacts their quality of life. To compensate, many individuals alter their diet by focusing on tastes, chemesthesis (e.g., chili pepper heat, menthol cooling), and the texture of foods to make it more palatable. Some diet alterations, such as increasing salt use, can result in a lower diet quality, and an increased risk for chronic disease. Sensory nutrition is an area of research that focuses on how the chemical senses (e.g. taste, smell, chemesthesis) and oral somatosensation) affect dietary choices and health. Sensory nutrition strategies designed for individuals with smell loss may help improve the flavor and liking of foods while improving diet quality, and are a necessary area of future research to help improve health and quality of life in the growing population with smell loss.

## Introduction

Prior to the COVID-19 pandemic, about 20% of people in the United States [1], Spain [2], Sweden [3], and Germany [4] had olfactory dysfunction, ensuing from a variety of etiologies such as traumatic brain injury, infection, sinonasal disease, cancer, and aging [1,5–7]. Now, as a cardinal symptom of the earliest variants of COVID-19 [8], smell loss has increased in prevalence. An estimated 20–67% of individuals with COVID-19 experience smell loss, depending on the SARS-CoV-2 variant [9,10]. This smell loss occurs in the form of hyposmia (i.e. reduced sense of smell) and anosmia (i.e. total smell loss; **H/A**). While most recover their sense of smell within 3 weeks [11–14], about 10–15% of individuals do not recover [11,15]. As a result, millions of individuals will now experience long-term smell loss because of COVID-19 [11,15].

Losing one’s sense of smell can be devastating to their eating enjoyment [16,17], diet quality [18,19], and quality of life [20]. Given that an unhealthy diet is associated with an increased risk for chronic diseases [21], understanding how to improve diet quality, particularly in the growing population with smell loss, is essential for health. Furthermore, aims to increase diet quality should also focus on palatability, as this is one of the strongest determinants of food choice [22]. Sensory nutrition is an area of research that focuses on how the chemical senses (e.g. taste, smell, chemesthesis), and oral somatosensation) affects dietary choices and health [23,24]. This area of research can help identify ways to improve diet quality and health by focusing on techniques to increase palatability of healthy foods. This mini-review will discuss how smell loss affects ingestive behavior, and highlights the need for more sensory nutrition research to improve diet quality while maintaining palatability in this population.

## The importance of our sense of smell in flavor perception

Flavor is a combination of the taste, smell, chemesthesis (i.e. the burning from peppers, the cooling of mint), and the oral somatosensation of foods and beverages [23]. Note that although the flavor of foods is often incorrectly referred to as the “taste” of food, taste only refers to the sensations of sweet, sour, salty, bitter, and umami (and perhaps fatty [25]) [26]. For the past decade, the flavor of foods has been rated the number one driver of food purchasing behavior, above price, healthfulness, convenience, and environmental sustainability [27], highlighting the importance of flavor in our lives. Smell is a fundamental component of flavor perception; if you eat an apple, you can taste the sweetness and sourness, and feel the crunch and the juice of the apple, but without smell, it would not have the apple flavor. In addition to its role in flavor perception, our sense of smell also plays a role in stimulating appetite for specific foods (i.e. sensory specific appetite) [28], helps to prepare our bodies to metabolically process foods (i.e. cephalic phase response) [29], and influences our food choices when perceived unconsciously (i.e. priming) [30], thereby affecting ingestive behavior. If someone loses their sense of smell, flavor perception, appetite, and ingestive behavior are likely to be affected.

## The effect of smell loss on food choice, nutrition, and chronic disease

One of the primary complaints of H/A individuals is that food is less flavorful and less enjoyable [17,19,31–33], which has profound adverse effects on their quality of life [17,20,34–37]. As a result, many of these individuals experience changes in their appetite and alter their diet to compensate for the lack of flavor [16,19,38–41]. One way H/A individuals compensate for flavor loss is by enhancing the tastes (i.e. sweet, sour, salty, bitter, umami) in their food [16]. In particular, many studies indicate that individuals self-report adding more salt to their foods after losing their sense of smell [17,42], and develop a preference for salty [16,36] and sweet foods [36]. Another way H/A individuals compensate for flavor loss is by adding spices or hot sauce to their foods to enhance the chemesthesis aspect of flavor [16,19,36,43]. For example, in a semi-structured interview conducted by Turner and Rogers, one participant noted: “…I opted for more spicy food…stuff with a lot more heat in it just to taste something” [36]. Lastly, individuals who lose their sense of smell also focus on palatable and contrasting textures and temperatures of their foods to try to enjoy eating again [33,36,43]. Foods that have a mushy or slimy texture are often unpleasant and avoided by those with smell loss, whereas those with crunchy textures tend to be more palatable [36]. The importance placed on these different dietary compensation strategies varies between cultures [44].

While not consistently reported [45–47], these alterations to make the diet more palatable may contribute to lower diet quality if they are maintained due to long-term smell loss. Individuals with smell loss from the Netherlands report poor appetite and a reduced diet quality compared to those with a normal sense of smell [48], and worse adherence to fiber, trans fatty acid, and alcohol recommendations compared to those with a normal sense of smell [46]. Data from the 2011–2014 National Health and Nutrition Examination Survey (NHANES) indicate that adults >40 years old with self-reported olfactory disorders have a significantly lower Healthy Eating Index 2015 (HEI-2015 score, which measures how closely the diet aligns with the 2015–2020 Dietary Guidelines for Americans), indicating a lower diet quality, compared to adults with a self-reported normal sense of smell [18]. The reduced diet quality in adults with olfactory dysfunction was attributed to lower consumption of total vegetables and greens and beans, and a higher consumption of added sugars and saturated fats compared to adults with a normal sense of smell. These differences in diet quality between adults with olfactory disorders and adults with a normal sense of smell were primarily driven by women aged 40–64, indicating that this age group may be especially important to target for any nutritional interventions due to smell loss. Women may be more likely to seek alterations in dietary choices than men because women are typically better at identifying odors, thus they may be more aware and attentive to the olfactory components of food flavor [49,50], and more affected by the loss.

The reduction in diet quality among H/A individuals may also increase their risk for diet-related disease. Data from the 2011–2014 NHANES also indicate that adults with olfactory disorder have a small but significantly higher BMI, waist circumference, and chronic disease score (calculated by adding whether someone has medically diagnosed diabetes, cancer, stroke, or heart attack) [18]. On the other hand, some studies have found that while some H/A individuals have clinically meaningful weight gain or weight loss, many maintain a stable body weight [38,51], indicating individual differences may exist. As seen with diet quality, differences between sex and age influence risk for diet-related disease. Data from the 2011–2014 NHANES indicate that women aged 40–64 with olfactory disorders have a higher BMI and waist circumference compared to those without olfactory disorders [52]. However, women 65 and older with olfactory disorders have lower total cholesterol and higher fasting glucose compared to those with a normal sense of smell [52]. For men 40–64 years old with olfactory disorders, they have lower fasting triglycerides and glucose compared to those with a normal sense of smell. Men 65 and older with olfactory disorders have a lower BMI, but higher total cholesterol and fasting LDL-cholesterol compared to those with a normal sense of smell [52]. These age and gender differences are likely due to differences in diet quality [52]. Thus, while olfactory disorders can affect risk for chronic disease, certain groups may be more prone than others to these deleterious effects.

## Importance of sensory nutrition in health

Despite the impact of smell loss on diet quality, disease risk, and quality of life, there is limited guidance for H/A individuals to improve their diet palatability and quality. Although charities such as AbScent [53], Fifth Sense [54], and The Smell and Taste Association of North America (STANA) [55] provide extensive resources and a sense of community for those suffering from smell disorders, the scientific basis for dietary guidance in these populations is lacking.

Sensory nutrition is an area of research that aims to understand how taste, smell, chemesthesis, and oral somatosensation affect dietary choices and health [23]. Sensory strategies are recommended to the population to improve diet quality while maintaining palatability in order to promote adherence to specific eating patterns. For example, the National Academy of Medicine (formerly known as the Institute of Medicine) recommends gradually reducing sensory exposure to salt (e.g., reducing exposure to the taste of salt) in order to prefer lower salt concentrations to reduce sodium intake [56–58]. Gradual reduction of sensory exposure to fat taste also results in lower pleasantness ratings for high-fat foods and a reduction in the preferred fat content of foods, which may promote adherence to a reduced-fat diet [59]. Congruent odors can also be added to foods to enhance tastes in order to increase palatability. For example, adding a vanilla aroma can enhance the sweetness of chocolate milk, although the effect is small and it may need to be combined with another strategy to help to reduce sugar intake [60]. However, whether these sensory strategies help to improve diet quality in individuals without a sense of smell is unknown, and a comparison of sensory strategies for those without a sense of smell has not been explored.

More research is needed to better understand how smell loss affects ingestive behavior, diet quality, and risk for chronic disease, especially given the growing population of those suffering from long-term smell loss because of COVID-19. Smell and taste loss also occur in the elderly and those with cancer, and research has focused on how eating behaviors are affected in these populations. However, changes in eating behavior may also be affected by side effects of aging (such as health disorders, medications, denture use, and oral hygiene [61]) and side effects of cancer treatments (such as dysphagia, dry mouth, nausea [62]) in addition to the taste and smell loss experienced by these populations. Furthermore, in cancer patients, taste changes, rather than smell loss, may drive eating behavior changes [62]. Thus, while these studies can help inform possible sensory strategies to improve flavor perception and diet quality, young and middle aged adults with smell loss but are otherwise healthy will also be an important population to study. Nevertheless, sensory strategies to improve flavor perception and eating enjoyment may be useful across all etiologies of smell dysfunction.

## Potential sensory strategies for individuals with smell loss

How can we improve flavor while we are still missing one of its biggest components, smell? One potential sensory strategy includes cross-modulation between taste and chemesthesis. For example, a low concentration of capsaicin (the spicy component of chili peppers) can lower the taste threshold for sweet, sour, salty, and bitter, indicating an increased sensitivity to these tastes [63]. There has been a focus on the cross-modulation between capsaicin and salt taste in particular, given that reducing sodium intake is a goal for several health organizations [64–67]. In those with a normal sense of smell, adding capsaicin to a salt solution can increase the perceived salt taste intensity [68], especially at low salt concentrations [69]. Therefore, low levels of capsaicin may help promote adherence to reduced sodium food products, and reduce salt intake, although additional research is needed to address this question. Studies are currently ongoing to determine if this phenomenon exists in H/A individuals [70]. Given that H/A individuals often report adding spice to foods to improve flavor, adding capsaicin to foods could be a useful strategy to reduce salt use while improving flavor perception and food liking. Low calorie sweeteners can be used to replace sources of nutritive sweeteners to reduce added sugar intake while maintaining sweet taste and palatability, and possibly improve diet quality depending on how they are incorporated into the diet [71]. However, some low calorie sweeteners have metallic or bitter tastes, which may create an unpleasant experience for those with smell loss relying on tastes for flavor [72,73]. Further studies are needed to identify sensory strategies to reduce added sugar and saturated fat intake, and increase vegetable intake in individuals with smell loss, which were identified as key areas affecting diet quality from previous studies [18]. Texture, in particular, could be a focus of increasing vegetable intake in individuals with smell loss, as crunchy textures (i.e. raw vegetables) tend to be more palatable than mushy textures (i.e. cooked vegetables) in those with smell disorders. Whether sensory strategies increase diet quality over time (through increased vegetable intake, or reduced salt, sugar, or fat intake), and whether they are robust enough to reduce risk factors for chronic disease will also be important future directions to improve the health of those suffering from smell loss.

## Conclusion

More people now have long-term smell disorders than ever before, which will likely negatively impact their quality of life and alter their eating habits in a way that reduces their diet quality. Current studies are ongoing to determine how capsaicin can increase salt taste intensity in those with smell loss, which could help to reduce salt intake and subsequent cardiovascular disease risk. Sensory nutrition strategies specifically in those with smell loss that target increasing flavor and food liking to improve diet quality and reduce the risk of chronic disease are a necessary area of future research.
